# Supplementary material for: A Magnetically and Electrically Powered Hybrid Micromotor in Conductive Solutions: Synergistic Propulsion Effects and Label‐Free Cargo Transport and Sensing
Source: Adv Sci (Weinh). 2022 Dec 11;10(8):2204931. doi: 10.1002/advs.202204931 (PMC10015886; doi:10.1002/advs.202204931)
Supplement: Supplementary file 1 — Supporting Information [file ADVS-10-2204931-s009.pdf]

ADVANCED SCIENCE
Open Access

Supporting Information

for *Adv. Sci.*, DOI 10.1002/advs.202204931

A Magnetically and Electrically Powered Hybrid Micromotor in Conductive Solutions: Synergistic Propulsion Effects and Label-Free Cargo Transport and Sensing

*Yue Wu, Sivan Yakov, Afu Fu and Gilad Yossifon**

Supporting Information

A Magnetically and Electrically Powered Hybrid Micromotor in Conductive Solutions: Synergistic Propulsion Effects and Label-Free Cargo Transport and Sensing

Yue Wu[1], Sivan Yakov[2], Afu Fu[3] and Gilad Yossifon[1,2]*

[1]School of Mechanical Engineering, University of Tel-Aviv, Tel-Aviv 69978, Israel

[2]Faculty of Mechanical Engineering, Micro- and Nanofluidics Laboratory, Technion – Israel Institute of Technology, Haifa 32000, Israel

[3]Technion Integrated Cancer Center, The Rappaport Faculty of Medicine and Research Institute, Technion-Israel Institute of Technology, Haifa 3109602, Israel

* Corresponding author: gyossifon@tauex.tau.ac.il

Supplementary Figures

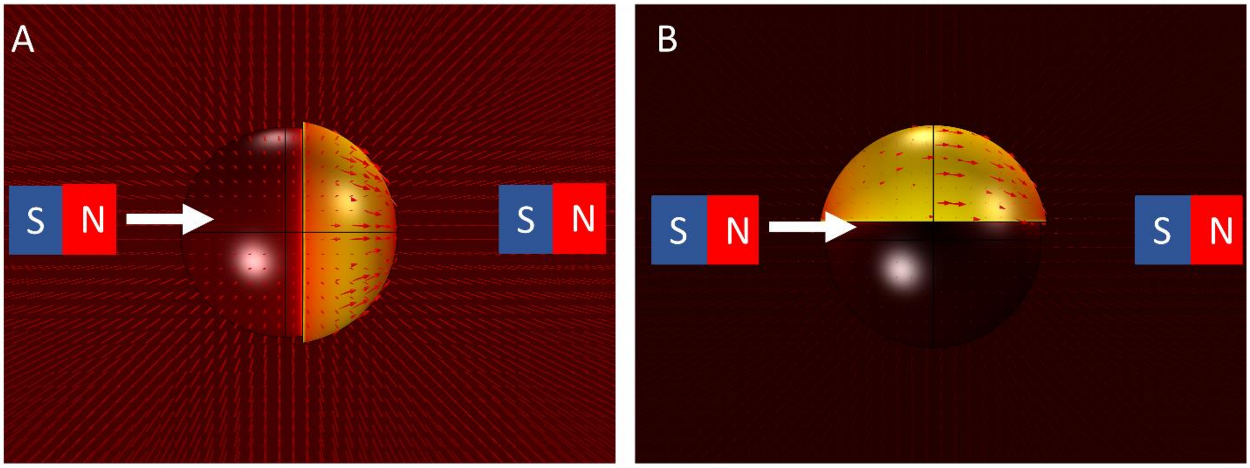

Figure. S1. Numerical simulations of the magnetic field vector obtained for magnetization (A) normal (as used in our previous publications[6],[7]) and tangential (as used in current setup (Fig. 1(A)) to the metallo-dielectric interface of the JP where the metallic coated hemisphere consists also of a ferromagnetic 50nm Ni layer sandwiched in between 15nm Cr and 15nm Au on the external layer.

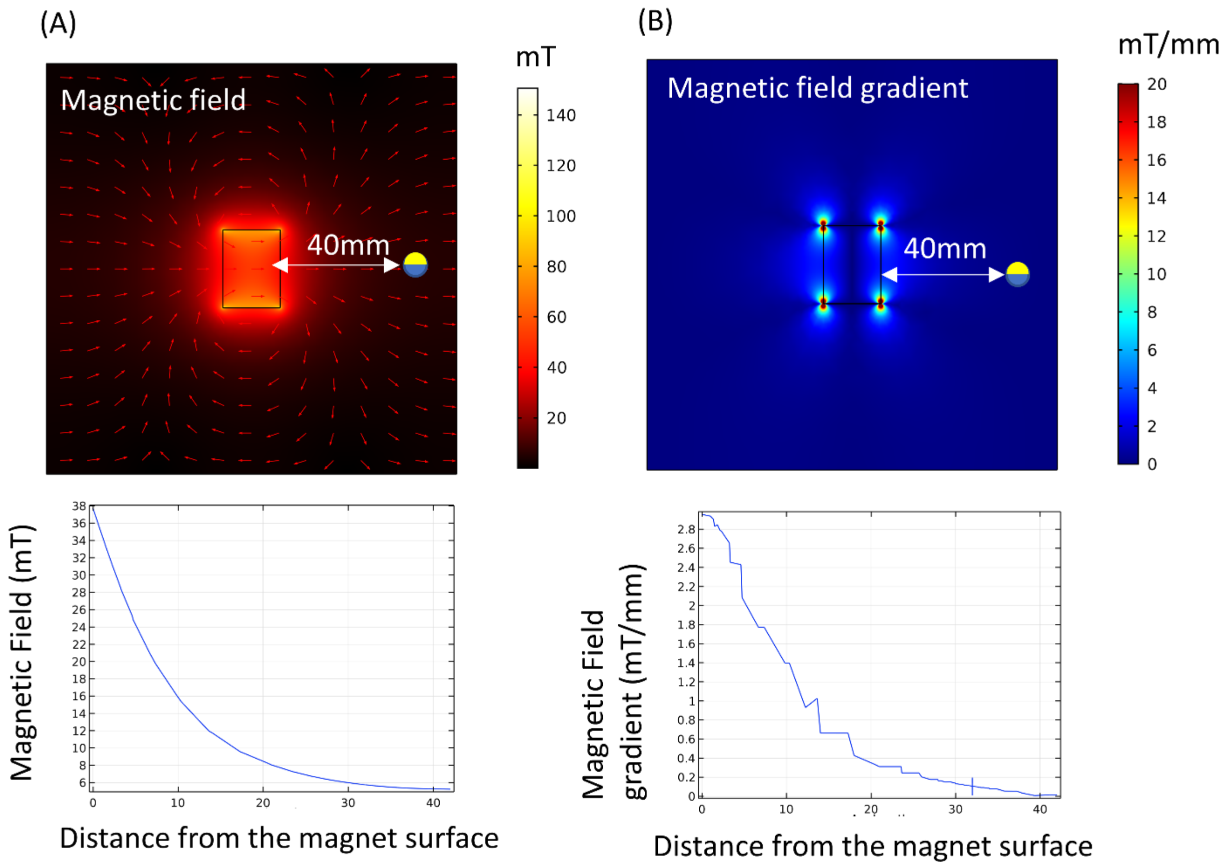

Fig.S2. The magnetic field vector (A) and gradient (B) induced by a block magnet and their corresponding spatial distribution along a horizontal line. The Janus particle in our experiments was located ~40mm away from the magnet's edge.

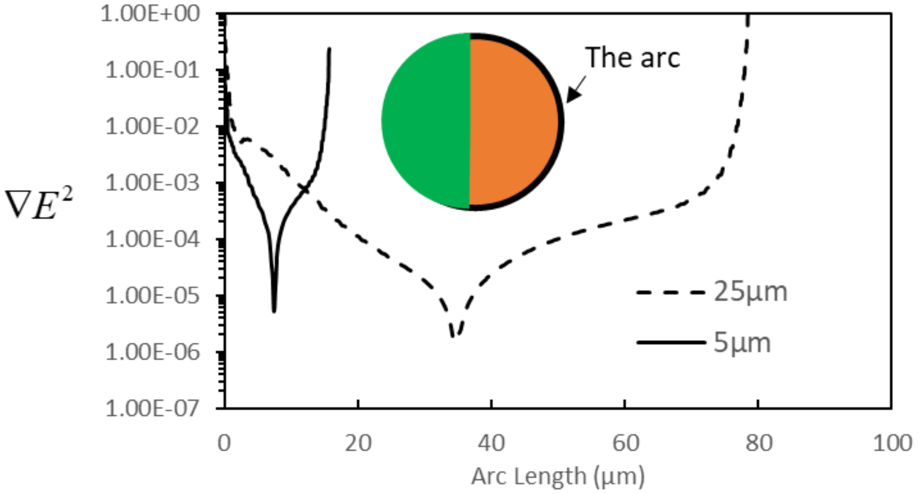

Fig. S3 Simulation of normalized DEP force ($\nabla E^2_{normalized} = \nabla E^2 / \nabla E^2_{\max}$) induced along the metallic coating of the Janus particle with diameter of 5 and 25μm.

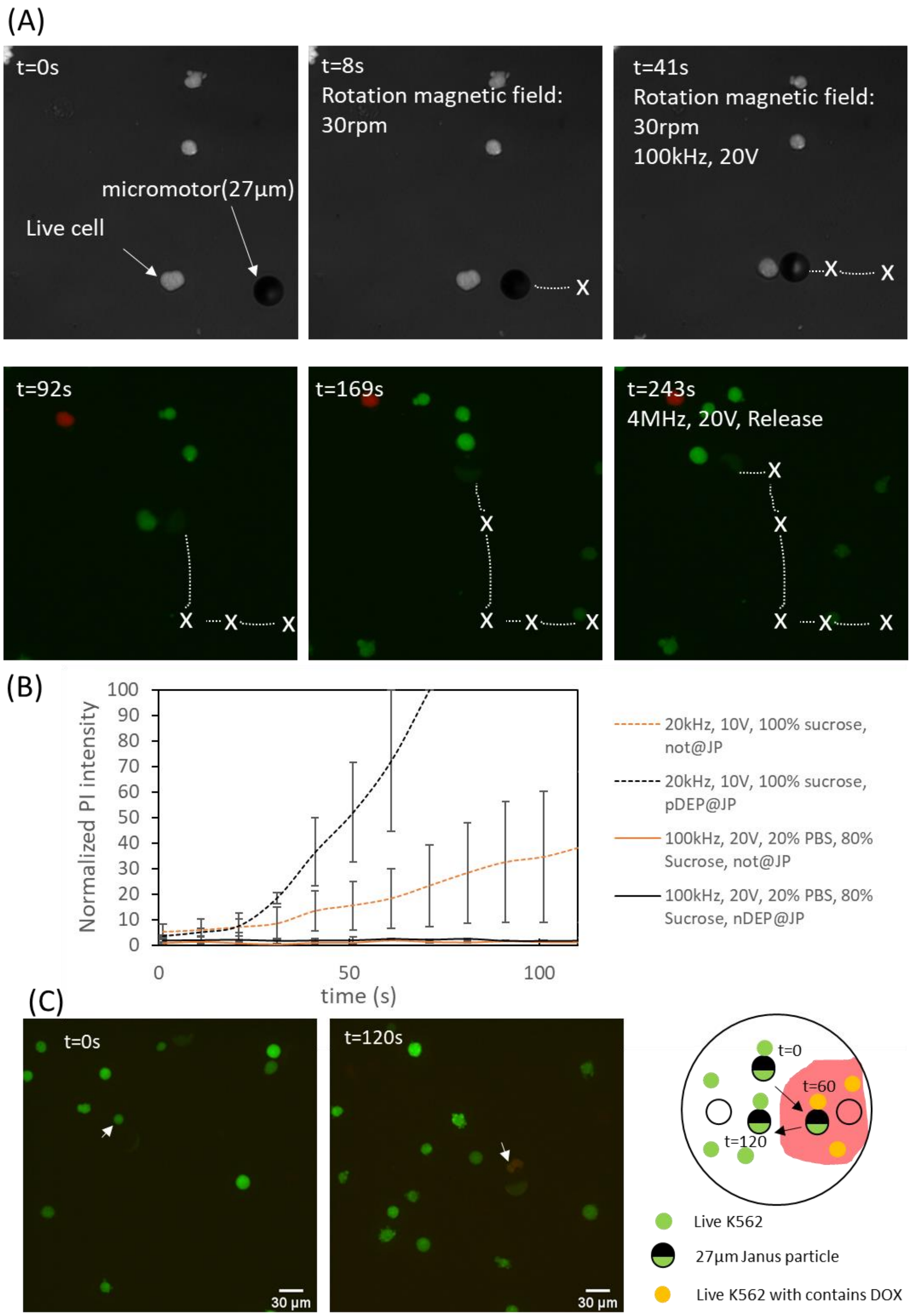

Figure. S4. Live K562 cell trapping and transport. (A) Sequential trapping and transport of intact K562 cells under 100kHz, 20V. (B) The trapped K562 cell under 100kHz, 20V remained intact for at least 120s. These experiments were performed within a 20% PBS, 80% sucrose solution (2.2-2.6mS/cm, pH: 7.31). (C) Transport of a live cell to (t=60s) and from (120s) a region containing doxorubicin (DOX)-loaded liposomes.

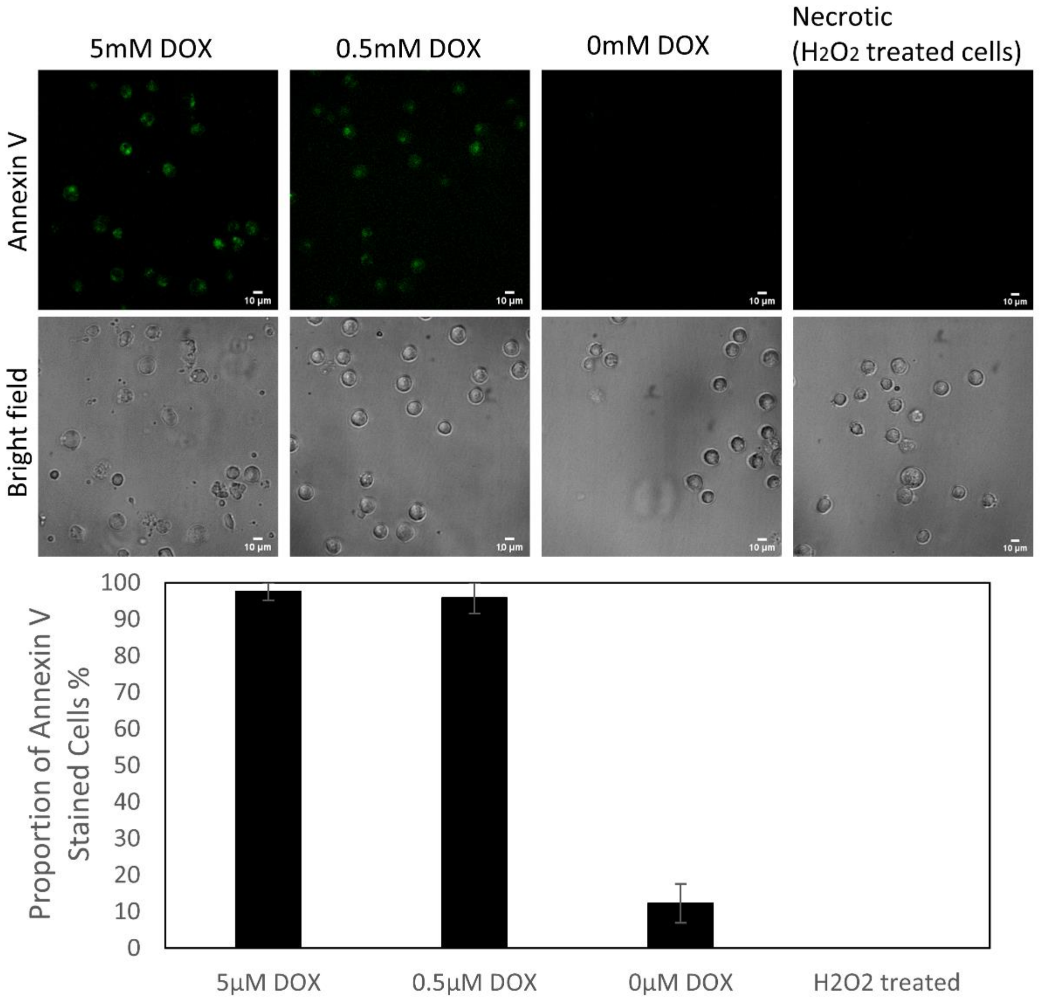

Figure. S5 Comparison of Annexin V staining of cells with different treatment: 5μM DOX (18hr), 0.5μM DOX (18hr), no DOX, and necrotic (H_2O_2 treated).

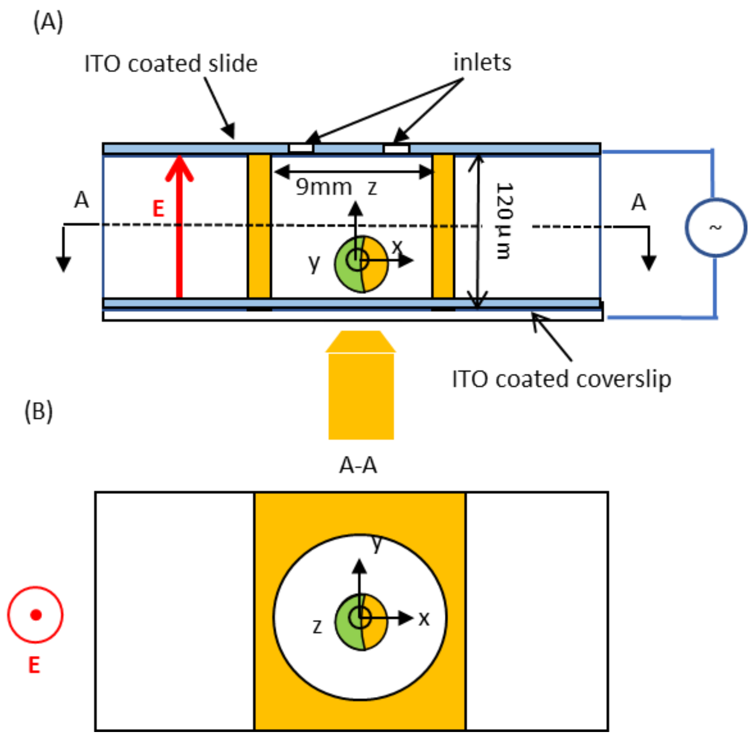

Figure. S6. Experimental setup. Two ITO-coated slides were separated by a 120 µm-thick silicone spacer. An external magnet was used to rotate the micromotor, while an electric field was used for micromotor alignment, electric propulsion and cargo manipulation. (A) Side view of the experimental chamber. (B) Cross section A−A, as visualized by the microscope.

Table S1. Numerical boundary conditions for Fig. 3G and Fig.4D.

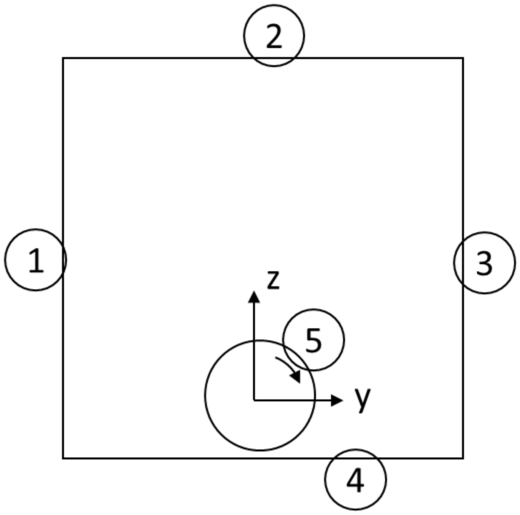

Boundary	conditions
1	Open boundary p=0
2	Open boundary p=0
3	Open boundary p=0
4	Moving wall
5	Rotating wall

Table S2. Dielectrophoretic Trapping conditions of various cargos by the JP

Cargo	Cargo diameter (μm)	Carrier (JP) size (μm)	Solution	Conductivity (mS/cm)	Solution pH	E-Field	DEP
Polystyrene sphere	15	27	0.001-0.01 M KCl	0.1-1.3	6.14	50kHz-1MHz 20V	nDEP
	10	27	0.001-0.01 M KCl	0.1-1.3	6.14	100kHz-1MHz 20V	nDEP
	5	10	0.001-0.01 M KCl	0.1-1.3	6.14	500kHz-1MHz 20V	nDEP
	3	5	0.001-0.005 M KCl	0.1-0.7	6.14	2MHz 20V	nDEP
Necrotic K562 (mammalian cell)	13-20	27	10% PBS, 90% 300mM sucrose	1-1.3	7.31	1MHz 20V	nDEP
			20% PBS, 80% 300mM sucrose	2.2-2.6	7.31	100kHz 20V	nDEP
5μM DOX treated K562 (mammalian cell)	13-20	27	10% PBS, 90% 300mM sucrose	1-1.3	7.31	100kHz 20V	nDEP
Intact K562 (mammalian cell)	13-20	27	20% PBS, 80% 300mM sucrose	2.2-2.6	7.31	100kHz 20V	nDEP
RBCs	Diameter: 7.5, thickness: 3	27	10% PBS, 90% 300mM sucrose	1-1.3	7.31	1MHz 20V	nDEP
			20% PBS, 80% 300mM sucrose	2.2-2.6	7.31	500kHz-1MHz 20V	nDEP
Rhodococcus (bacterial cell)	1-2	5	10% PBS, 90% 300mM sucrose	1-1.3	7.31	6MHz 20V	pDEP

Supplementary videos

Video 1. Manipulation of a microbot by both electric field and external rotation magnetic field.

Video 2. Two scenarios showing low oscillation factor and high oscillation factor.

Video 3. The influence of magnetic rolling of the JP on its electric field velocity ($V_{electric}$) in conductive solution.

Video 4. Trapping and transport of a polystyrene (PS) particle, red blood cell (RBC) and bacteria.

Video 5. Selective trapping of a necrotic K562 cell in 10% PBS, 90% 300mM sucrose, under 1MHz, 20V.

Video 6. Manipulating late, early apoptotic and viable cells within 10% PBS, 90% 300mM sucrose under 100kHz, 20V.

Video 7. Trapping and transport of an intact and live K562 cell in 20% PBS, 80% 300mM sucrose, under 100kHz, 20V.

Video 8. Trapping and transport of an intact and live K562 cell in 1x PBS at 1MHz, 75V.
